# Discovery of a Series of Acridinones as Mechanism-Based Tubulin Assembly Inhibitors with Anticancer Activity

**DOI:** 10.1371/journal.pone.0160842

**Published:** 2016-08-10

**Authors:** Luma G. Magalhaes, Fernando B. Marques, Marina B. da Fonseca, Kamilla R. Rogério, Cedric S. Graebin, Adriano D. Andricopulo

**Affiliations:** 1 Laboratório de Química Medicinal e Computacional, Centro de Pesquisa e Inovação em Biodiversidade e Fármacos, Instituto de Física de São Carlos, Universidade de São Paulo, 13563–120, São Carlos-SP, Brazil; 2 Laboratório de Diversidade Molecular e Química Medicinal, Departamento de Química, Universidade Federal Rural do Rio de Janeiro, 23897–000, Seropédica-RJ, Brazil; Columbia University, UNITED STATES

## Abstract

Microtubules play critical roles in vital cell processes, including cell growth, division, and migration. Microtubule-targeting small molecules are chemotherapeutic agents that are widely used in the treatment of cancer. Many of these compounds are structurally complex natural products (e.g., paclitaxel, vinblastine, and vincristine) with multiple stereogenic centers. Because of the scarcity of their natural sources and the difficulty of their partial or total synthesis, as well as problems related to their bioavailability, toxicity, and resistance, there is an urgent need for novel microtubule binding agents that are effective for treating cancer but do not have these disadvantages. In the present work, our lead discovery effort toward less structurally complex synthetic compounds led to the discovery of a series of acridinones inspired by the structure of podophyllotoxin, a natural product with important microtubule assembly inhibitory activity, as novel mechanism-based tubulin assembly inhibitors with potent anticancer properties and low toxicity. The compounds were evaluated *in vitro* by wound healing assays employing the metastatic and triple negative breast cancer cell line MDA-MB-231. Four compounds with IC_50_ values between 0.294 and 1.7 μM were identified. These compounds showed selective cytotoxicity against MDA-MB-231 and DU-145 cancer cell lines and promoted cell cycle arrest in G_2_/M phase and apoptosis. Consistent with molecular modeling results, the acridinones inhibited tubulin assembly in *in vitro* polymerization assays with IC_50_ values between 0.9 and 13 μM. Their binding to the colchicine-binding site of tubulin was confirmed through competitive assays.

## Introduction

Microtubules are cytoskeletal protein polymers involved in essential cellular processes such as cell migration, intracellular transport and mitosis. They are a dynamic system composed of α and β tubulin heterodimers, which are assembled in the so-called fast-growing plus-end and disassembled from the slow-growing minus-end [1–6]. Suppression of this dynamic instability interferes with microtubule functions, including mitotic spindle formation. The disruption of mitotic spindle formation blocks mitosis and arrests the cell cycle in the G_2_/M phase, leading to apoptosis [2, 5, 7]. Tubulin is therefore considered one of the most important anticancer targets. In addition, it is modulated by clinically relevant compounds such as paclitaxel, vinblastine and vincristine [8, 9]. Microtubule-targeting agents are classified into two categories according to their mechanism of action: (i) microtubule-stabilizing agents, which mostly bind to the tubulin paclitaxel-binding site, and (ii) microtubule-destabilizing agents, which usually bind to the tubulin vinblastine-binding or colchicine-binding sites [10].

Despite the clinical relevance of these drugs, serious problems with pharmacokinetics, toxicity, and resistance limit their therapeutic usefulness [5, 11–13]. The natural product colchicine, an interesting tricyclic alkaloid microtubule-destabilizing agent that binds to the colchicine-binding site of tubulin, is not suitable for therapeutic use owing to its high toxicity profile [14]. The taxanes (e.g., paclitaxel) and vinca alkaloids (e.g., vinblastine, and vincristine), natural products that modulate tubulin assembly, are structurally complex compounds containing several stereogenic centers [12, 13], making them difficult to synthesize. Moreover, it is important to consider the scarcity of some of the natural resources used in manufacturing drugs. Therefore, there is a great deal of interest in the development of novel, structurally simple, easily synthesized, microtubule-binding antimitotic agents to overcome these limitations. The discovery of naturally occurring combretastatin A-4 as an inhibitor of tubulin polymerization with potent cytotoxic activity has reinforced the importance of developing clinically relevant colchicine-binding site inhibitors (CBSIs). [15, 16].

Podophyllotoxin, a structurally complex toxin lignan obtained from plants of the *Podophyllum* genus, is another important ligand that binds to the colchicine-binding site with remarkable microtubule assembly inhibitory activity, but its therapeutic use has been restricted because of its high toxicity. Here, we describe our lead discovery approach inspired by the structure of podophyllotoxin. This strategy, based on the synthesis and evaluation of compounds with low structural complexity that mimic the colchicine-binding site properties of the natural molecule, enabled the identification of an interesting series of synthetic acridinones as novel CBSIs with potent anticancer activity and low toxicity. The chemical structures of colchicine, paclitaxel, vinblastine, vincristine, combretastatin A-4 and podophyllotoxin are shown in S1 Fig.

## Materials and Methods

### Chemical synthesis

To obtain the designed acridin-8-one derivatives, we employed the Hantzsch-based multicomponent protocol developed by Husson *et al* [17]. A mixture of three reagents (aromatic aldehyde, aromatic amine and 1,3-cyclohexanedione) were reacted in a one-pot process, which generated the desired heterocyclic compounds (**1–15**, S1 Appendix) as products.

### Molecular modeling

Three-dimensional (3D) structures were generated using a sketch tool and the Tripos force field, with charges computed by the Geisteiger Hückel method. The modeling studies were executed on the molecular modeling platform SYBYL-X (Tripos, St. Louis, MO, USA). Docking studies were conducted using the three known tubulin-binding sites (colchicine, vinblastine and taxol). The X-ray crystal structures in complex with the three modulators—DAMA-colchicine, vinblastine and taxol (PDB IDs 1SA0, 1Z2B and 1JFF, respectively)—were obtained from the Protein Data Bank. For the simulations, the ligands were removed and hydrogen atoms were added. Simulations were performed using Surflex-Dock [18] (Tripos, St. Louis, MO, USA), Gold 5.2 [19] (Cambridge Crystallographic Data Centre, Cambridge, UK) and Autodock Vina [20] (Scripps Research Institute, San Diego, CA, USA) docking programs. The original ligand coordinates were used as a reference to locate the docking site in all cases.

The Surflex program was set using standard parameters. When Gold was used, the taxol and vinblastine sites were defined as the residues within a sphere with a 12 Å radius around the original ligand; for the colchicine cavity, this site was considered to have an 8 Å radius. The molecules were scored for their receptor complementarity based on the sums of their van der Waals and hydrogen interaction energies using the GoldScore function. For the calculations, the targets were kept rigid but torsional flexibility was permitted for ligands. For Autodock-Vina, a configuration file was generated to determine the docking site as the residues inside a cubic box centered in the original ligand, with a 20 Å edge for taxol and vinblastine and a 15 Å edge for colchicine. The grid space was 0.375 Å. All the methods were validated by redocking the original ligand.

### Cell culture and lines

All the cell lines were purchased from the Rio de Janeiro Cell Bank (BCRJ, Rio de Janeiro, Brazil). The human breast cancer cell line MDA-MB-231 was maintained in L-15 (Leibovitz) supplemented with 10% FBS at 37°C in a humidified atmosphere. The human prostate cancer cell line DU-145 was grown in RPMI (RoswellPark Memorial Institute), and the human fibroblast cell line FGH was grown in Dulbecco’s Modified Eagle Medium. Both were supplemented with 10% FBS and maintained at 37°C in a 5% CO_2_ humidified atmosphere. Cell viability was assessed using Trypan blue exclusion before the experiments.

### Wound healing assay

To qualitatively evaluate directional cell migration [21, 22], MDA-MB-231 cells were seeded at a density of 1 x 10^5^ cells/well in 24-well culture plates (TPP^TM^) and allowed to reach a confluent monolayer. Then, the wells were scratched using a sterile 100 μL pipette tip. The medium was removed and fresh medium without FBS was added to clean the debris. Next, the cells were incubated for 22 h at 37°C with supplemented medium (10% FBS) and different concentrations of test compounds (10 μM, 1 μM and 0.250 μM). When measuring the wound closure, the wells were photographed under a 4X objective (Optiphase) at 0 and 22 h. The images were analyzed using Image J software, and two independent experiments were conducted in triplicate.

### Boyden chamber migration assay

Transwells containing 0.3 cm^2^ inserts with 8.0 μm pore polyethylene terephthalate membranes (Corning Falcon^TM^) were used to quantify cell migration [23]. MDA-MB-231 cells were added at a density of 4 x 10^4^ cells/insert in serum-free media (300 μL) in the upper chambers. In the lower chambers, 700 μL of medium supplemented with 10% FBS was added. Test compounds were added at 7 different concentrations (10–0.04 μM) in both the upper and lower chambers. The systems were incubated at 37°C for 6 h. Next, the cells inside the insert were removed with a cotton swab and the cells under the membrane were fixed with methanol and stained with toluidine blue for 5 min. Pictures of the stained membrane were taken under a 10X objective (Optiphase), and the cells were quantified using NIS Elements software (NIKON). Two independent experiments were conducted in duplicate.

### Cytotoxicity assay

MDA-MB-231, DU-145 and FGH cell lines were plated at a concentration of 4 x 10^3^ cells/well in 96-well culture plates (TPP^TM^). After 24 h, 10 concentrations (100–0.16 μM) of the test compounds were added in triplicate to the wells, and the plates were incubated for 72 h at 37°C in a 5% CO_2_ humidified atmosphere (if necessary). Then, 20 μL of the MTS reagent [24] (CellTiter 96^®^ AQueous One Solution Cell Proliferation Assay, Promega) was added to the wells, and the cells were incubated for 3 to 4 h at 37°C. Next, absorbance was measured at 490 nm using a spectrophotometer (SpectraMax Plus384, Molecular Devices). The percentage of unviable cells was determined in relation to the control wells, and at least two independent experiments were carried out for each test compound.

### Fluorescence-based tubulin polymerization assay

Tubulin polymerization assay [25] kit BK011P (Cytoskeleton) was used according to the manufacturer’s instructions. Briefly, 5 μL aliquots of the test compounds solubilized in 10% DMSO were added (in triplicate) to the wells of a 96-well plate (OptiPlate-1536 F–Black, PerkinElmer). Then, 45 μL of the reaction mixture (2 mg/mL tubulin (> 99% pure), 80 mM PIPES, 0.5 mM EGTA, 1 mM GTP, 2 mM MgCl_2_, glycerol 15% and 10 μM DAPI, pH 6.9) was added to each well, and the plate was immediately placed in a fluorimeter (Victor3, PerkinElmer). Fluorescence at 355_Ex_/460_Em_ was measured every 90 s for 1 h. To determine IC_50_, we applied 7 concentrations of the test-compounds ranging from 0.256 to 50 μM. The curves were normalized, and the percentage of inhibition was calculated by comparing the polymerization reaction at 25 min relative to vehicle control.

### Light-scattering-based tubulin polymerization assay

To independently confirm the ability of the compounds to inhibit tubulin assembly, a second tubulin polymerization assay was carried out [26]. We added 20 μL aliquots of the test compounds solubilized in 10% DMSO (in triplicate) to the wells of a 96-well culture plate (TPP^TM^). Then, 180 μL of 3 mg/mL tubulin (>97% pure, HTS03, Cytoskeleton) in G-PEM buffer (80 mM PIPES, 1 mM EGTA, 2 mM GTP, 1 mM MgCl2 and glycerol 5%, pH 7,0) was added to the wells, and the absorbance at 340 nm was measured every 40 s for 1 h using a SpectraMax Plus384.

### Colchicine-site competitive assay

The reaction mixture contained 1960 μL of 1 mg/mL tubulin (>97% pure, HTS03, Cytoskeleton) in 0.25 mM PIPES, 0.05 mM GTP and 0.25 mM MgCl_2_ at pH 6.9. The reaction mixture was incubated at 37°C with DMSO (negative control), 20 μL of colchicine (positive control, 5 mM stock) or 20 μL of a test compound (5 mM stock) for 30 min. After which, 20 μL of fluorescent colchicine (50 μM stock) was added and the reaction was incubated for 30 min at 37°C. Then, the solution was applied to a Sephadex G25 fine chromatography column 5 cm in length, and 150 μL of the eluate was collected in a 96-well plate (OptiPlate-1536 F–Black, PerkinElmer). When 24 wells were filled, the plate was read using a fluorimeter (Victor3, PerkinElmer) at 485 nm excitation and 535 nm emission wavelengths [27].

### Cell-cycle analysis

MDA-MB-231 cells were added at a density of 5 x 10^5^ cells in 25 cm^2^ culture bottles (TPP^TM^) with 5 mL of medium supplemented with 10% FBS. After 36 h, the media was removed, and 5 mL of fresh media without FBS was added. The bottles were then incubated for 48 h for cell-cycle synchronization [28]. Then, the cells were treated with the test compounds (1 or 3 μM) for 48 h. Subsequently, the cells were harvested by trypsinization, and their chromatin was fixed and stained following the manufacturer’s instructions (cat. no. 340242, BD Biosciences^TM^) [29, 30]. Shortly thereafter, the cells were centrifuged at 300 rcf for 5 min and resuspended in 1 mL of buffer (sodium citrate, sucrose, and DMSO) three times. From this suspension, 1 x 10^6^ cells were centrifuged at 400 rcf for 5 min, resuspended in 250 μL of solution A (trypsin in a spermine tetrahydrochloride detergent buffer) and incubated for 10 min at room temperature. Next, 200 μL of solution B (trypsin inhibitor and ribonuclease A in citrate-stabilizing buffer with spermine tetrahydrochloride) was added, and the suspension was incubated for 10 min at room temperature. Finally, 200 μL of solution C (propidium iodide (PI) and spermine tetrahydrochloride in citrate stabilizing buffer) was added, and the sample was incubated for 15 min in the refrigerator. The samples were then analyzed by flow cytometry using a BD Accuri C5 (BD Biosciences^TM^) system (excitation at 488 nm and emission detected with a 585/40 nm filter).

### Apoptosis analysis

Apoptosis was measured by flow cytometry via double staining with FITC-Annexin V and PI (cat. no. 556570, BD Pharmigen^TM^) [31]. Cells were prepared in the same way as described for the cell-cycle analysis, except that the cell-synchronization step was omitted in this case. After treatment with the test compounds for 24 or 72 h, the cells were harvested by trypsinization and centrifuged for 10 min at 150 rcf. The pellet was resuspended in annexin V binding buffer at a concentration of 10^6^ cells/mL. Then, 100 μL of this suspension was collected and stained with 5 μL of PI and FITC-Annexin V for 15 min at room temperature in the dark. After this, 400 μL of annexin V binding buffer was added, and the samples were analyzed using a BD Accuri C5 cytometer with excitation at 488 nm and emission detected with 533/30 nm (FITC) and 670 LP nm (PI) filters.

### Statistical analysis

Bar graphs and mean values ± SD were calculated and plotted using Graphpad Prism 6 (Graphpad Software). Two-tailed *t*-tests were conducted. * *P* <0.05, ** *P* <0.01 and *** *P* <0.001 were considered significant. The IC_50_ values were calculated using nonlinear regression analysis with Sigmaplot 10.0 (Systat Software).

## Results

### Drug design approach and chemical synthesis

**Fig 1:**
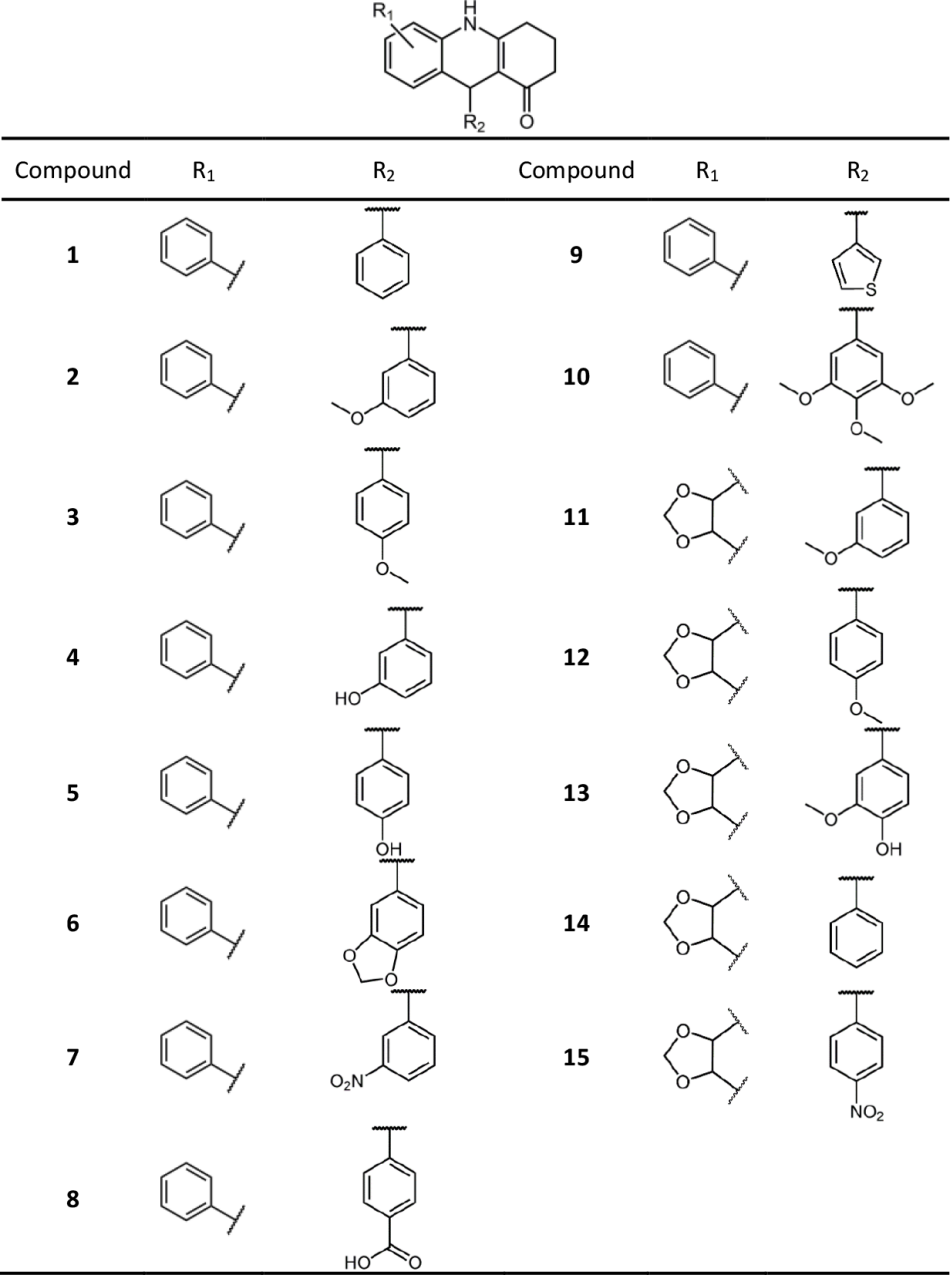
Structures of the synthetic acridinones.

Extensive efforts to reduce the toxic effects of podophyllotoxin have led to the development of etoposide and teniposide as novel anti-cancer drugs [32]. These chemotherapeutics are semisynthetic derivatives of podophyllotoxin that possess higher structural complexity. In the present study, our research approach employed medicinal chemistry lead discovery approaches to the development of less structurally complex podophyllotoxin derivatives possessing colchicine-binding site ligand properties, microtubule assembly inhibitory activity, anticancer activity and low toxic effects. In this context, reports have described the synthesis of aza-analogues of podohyllotoxin in a one-pot multicomponent procedure [17] employing 3,4,5-trimethoxybenzaldehyde, methylenedioxoanilin and tetronic acid as components. Recently, the scope of this reaction was expanded to various aromatic aldehydes and aromatic/heterocyclic amines [33]. Based on our early molecular modeling investigations, we explored the versatility of this reaction by synthesizing a collection of compounds employing naphthylamines, aromatic aldehydes and 1,3-cyclohexanedione as building blocks to verify the effects of ring expansion to a 6-member ring (instead of the original 5-membered one) and substitution of the heteroatom oxygen by a methylene (-CH_2_-) group (S2 Fig.). This strategy has some key advantages. One is that the final product is obtained in a single synthetic step, without the need to purify the reaction intermediates. Another advantage is that the obtained aza-analogs, although very similar to podophyllotoxin, have simplified molecular frameworks regarding the number of stereocenters—there is only a single chiral center instead of the four consecutive chiral centers of podophyllotoxin, as can be seen in our small library of synthetic compounds (Fig 1).

### Molecular modeling

The tubulin cavities of paclitaxel, vinblastine and colchicine were carefully investigated via molecular docking studies. The interaction of small molecules with the paclitaxel-binding site has been shown to stabilize the M-loop of β-tubulin into a helix that promotes lateral interaction between the protofilaments [34]. In the current study, the acridinones docked into the paclitaxel site were not able to properly interact with this loop and presented low structural complementarity with this binding site because these molecules are too small to fit inside a rather large binding pocket. Molecules that bind to the vinblastine-binding site interact with the α- and β-tubulins of two heterodimers, inducing a curved protofilament conformation that prevents tubulin polymerization [35]. The acridinones do not interact effectively within this large pocket, as is similarly observed for the paclitaxel-binding site.

By contrast, the colchicine-binding site, located at the tubulin α/β interface, accommodates the trimethoxyphenyl moiety of colchicine in a hydrophobic pocket composed of Leuβ248, Leu β252 and Leu β255 from the βT7 loop and βH8 helix, whereas the middle methoxy group (of the three methoxy groups) forms a hydrogen bond with Cysβ241 of the βH7 helix [36]. Colchicine binding leads to a curved tubulin dimer that avoids the formation of a straight structure and inhibits microtubule polymerization. This is due to the steric hindrance between colchicine and Asnα101 and Valα181 of the αT5 loop, as well as to changes in the βT7 loop and βH8 helix positions [37].

**Fig 2:**
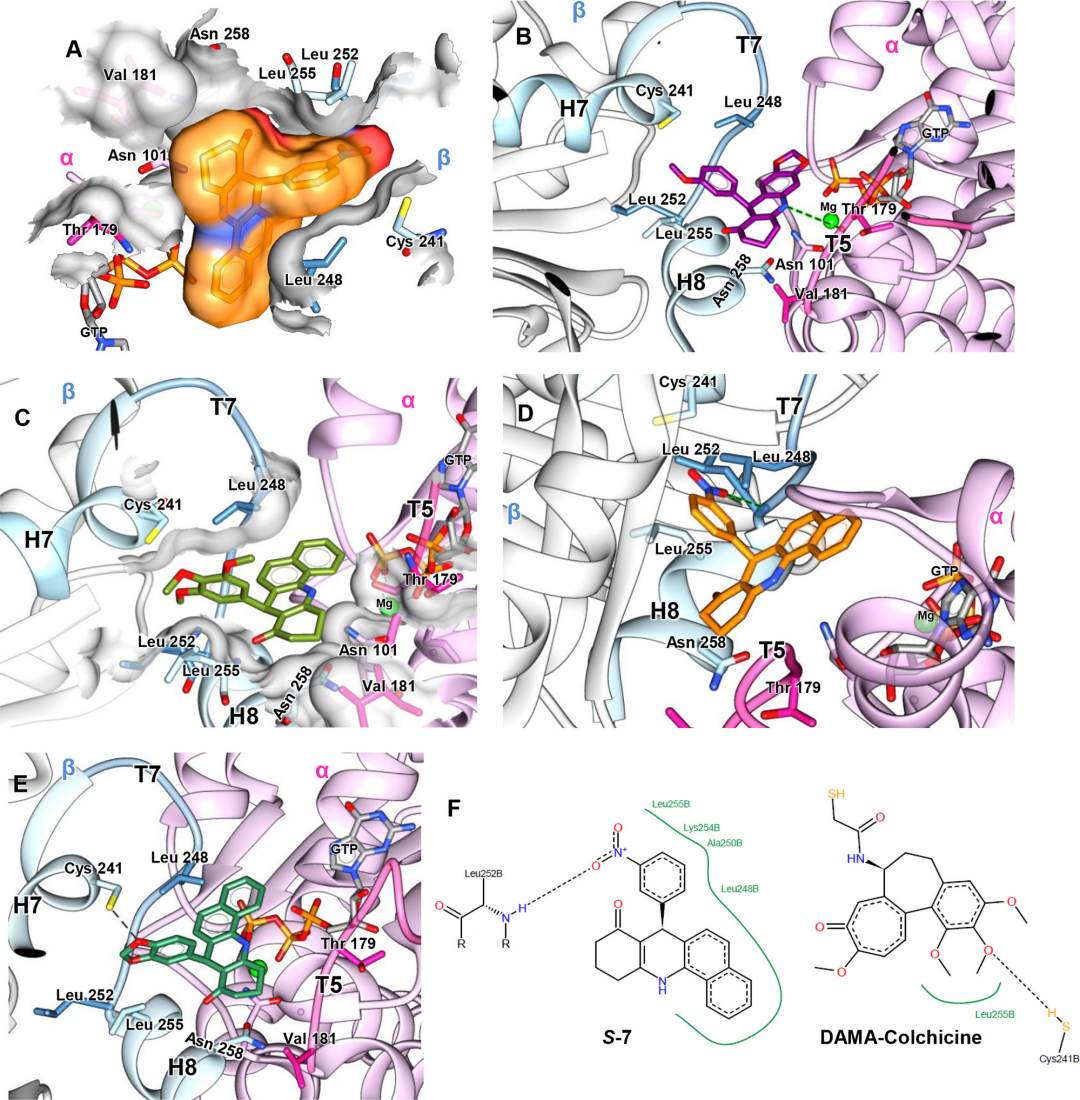
Molecular modeling studies of the acridinones series. (A), steric complementarity between the colchicine site and ***S*-7** (B), binding mode of ***S*-11** showing a hydrogen bond between the NH groups of acridinones and Thr α179 (C), binding mode of ***S*-10** where the trimethoxybenzyl group occupies the hydrophobic pocket of the colchicine site (D), docking pose for ***S*-7** with a hydrogen bond between the NO_2_ group and Leu β252 (E), docking pose for ***S*-6** with a hydrogen bond between oxygen from dioxalane and Cys β241 (F), Comparison of predicted interactions between the docking pose of ***S*-7** and the crystal structure of tubulin (PDB ID: 1SA0) and the predicted interactions between crystallographic DAMA-colchicine (PDB ID: CN2) and tubulin (PDB ID: 1SA0). These interactions were calculated using the online software PoseView [38]. The green lines represent hydrophobic interactions and the black dotted lines represent hydrogen bonds.

In the current study, the molecular modeling analysis in the colchicine-binding pocket indicated high structural complementarity with the acridinone scaffold docked between the amino acids Valα181, Thrα179 and Asnβ258 (Fig 2A). For instance, compounds such as ***S*-11** were able to establish a hydrogen bond between the residue Thrα179 and the NH group of the ligand (Fig 2B), similar to what was observed between Thrα179 and the OH group of podophyllotoxin. These are favorable interactions, considering that these residues compose the αT5 loop and βH8 helix, whose steric hindrance prevents tubulin from adopting a straight structure. Furthermore, the difference in binding of the *R*- and *S*-enantiomers was significant, suggesting that the *S*-enantiomers were in more favorable conformation for interacting with this loop. The phenyl-substituted acridinones were preferentially located in the same hydrophobic pocket of the trimethoxyphenyl group of colchicine and podophyllotoxin, thus preventing the βT7 loop and βH8 helix from assuming straight heterodimer positions (Fig 2C). Hydrogen bonds between the NO_2_ group of ***S*-7** and Leuβ252 and those between the O in the dioxolane heterocyclic of ***S*-6** and Cysβ241 (Fig 2D and 2E) were also identified. Additionally, this molecule allowed more favorable hydrophobic interactions with the pocket than those with colchicine (Fig 2F). Thus, the molecular modeling studies revealed that the acridinones and the colchicine-binding pocket have a higher level of interaction and structural complementarity, which are key elements for molecular recognition and consequently for inhibition of tubulin polymerization.

### Wound healing assay

**Fig 3:**
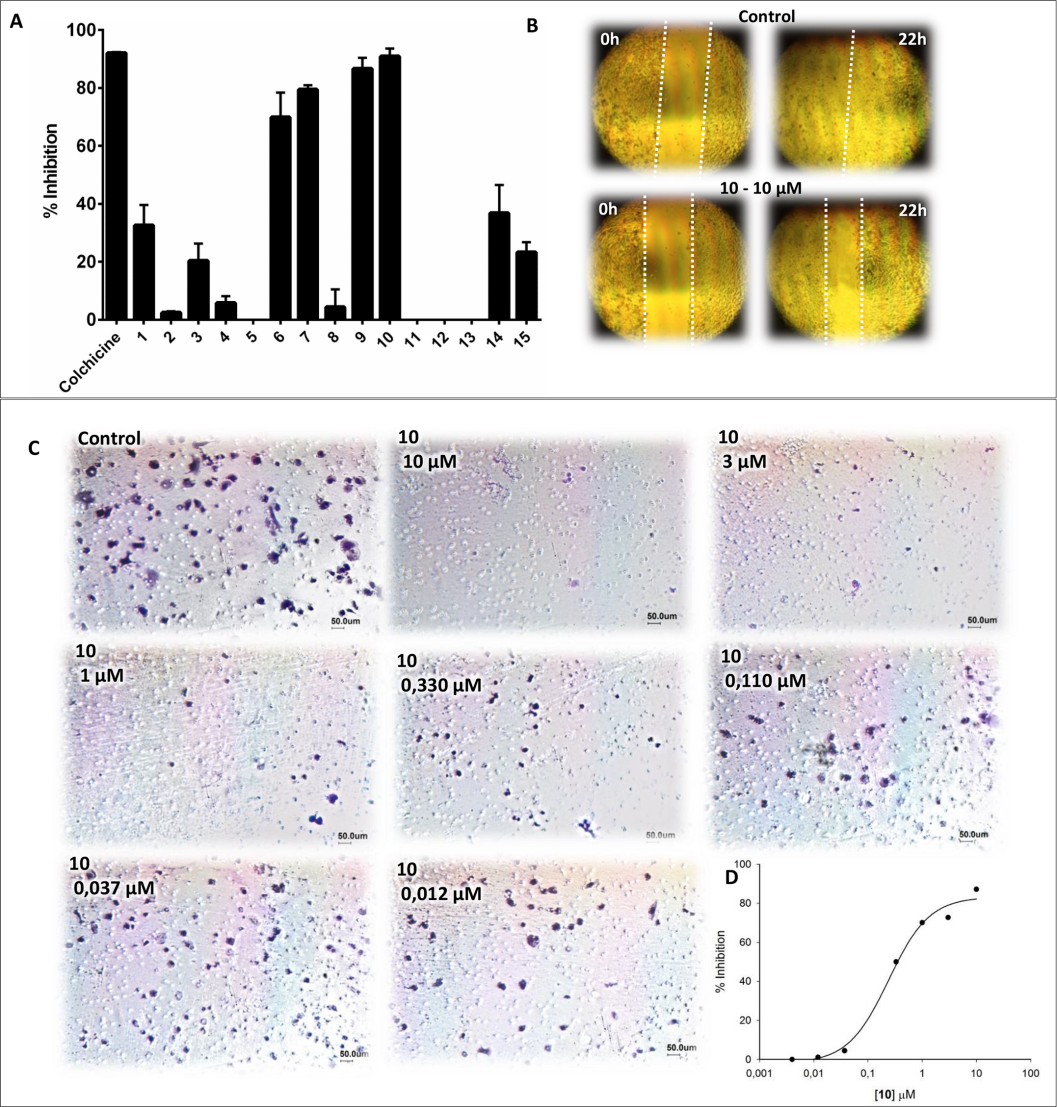
Effects of the acridinones on MDA-MB-231 cell migration. (A), inhibition of wound closure (concentration of 10 μM) (B), pictures of the wound healing assay at 0 h and 22 h for the negative control and **10** (C), representative pictures of migrated cells in a Boyden chamber assay for the negative control and **10** in different concentrations (D), a representative IC_50_ curve for the antimigratory response against **10**.

The molecular modeling results encouraged us to study the binding properties of this series of acridinones to tubulin. The compounds would be expected to have anti-migratory and cytotoxic effects because cell polarization is maintained by microtubules and is necessary for directional cell migration. Accordingly, we initially performed a wound healing assay to investigate the anti-migratory properties of these compounds. Using colchicine as a positive control, all the compounds were screened at 10 μM, which led to the identification of a subset of acridinones (**6**, **7**, **9** and **10)** with the ability to inhibit cell migration (Fig 3A and 3B).

### Boyden chamber migration assays

Following the qualitative analyses (i.e., wound healing assays), we dealt with the quantitative characterization of the anti-migratory effects of the active acridinones through concentration-response transwell migration assays. Compounds **6**, **7**, **9** and **10** exhibited IC_50_ values ranging from 0.294 to 1.345 μM (Table 1). Remarkably, compounds **7** and **10** had potency (IC_50_ values of 0.294 and 0.330 μM, respectively) comparable to that of colchicine (IC_50_ value of 0.320 μM), indicating the high potential of these compounds. It is worth noting that **10** possesses the same 3,4,5-trimethoxyphenyl moiety present in the structures of colchicine and podophyllotoxin, which are important ligands of the colchicine-binding site of tubulin [7]. The concentration-response effects of **10** on cell migration are depicted in Fig 3C and 3D.

**Table 1:** Inhibition of cancer cell migration, cytotoxicity and tubulin polymerization.

Compound	IC_50_ (μM)					
	Cell migration^a^	Cytotoxicity^b^				Tubulin assembly
		MDA-MB-231	DU-145	FGH	SI^c^	
**Colchicine**	0.320 ± 0.003	0.02 ± 0.01	0.025 ±0.003	< 0.02	1	0.99 ± 0.01^d^
**6**	1.7 ± 0.7	3 ± 1	3.2 ± 0.7	> 100	> 30	13 ± 2^d^
**7**	0.294 ± 0.005	0.190 ± 0.007	0.308 ± 0.05	> 100	> 100	2.4 ± 0.2^d^
**9**	1.345 ± 0.007	1.1 ± 0.2	1.0 ± 0.3	> 100	> 100	5 ± 2^e^
**10**	0.33 ± 0.02	0.11 ± 0.02	0.12 ± 0.02	> 100	> 100	0.9 ± 0.2^d^

^a^ Averages ± SD of two independent experiments conducted in triplicate.

^b^ 72 h MTS assay. Data are the averages ± SD of two independent experiments conducted in triplicate.

^c^ Selectivity index (SI = IC_50_^MDA-MB^ / IC_50_^FGH^)

^d^ Fluorescence assay. Data are the average of triplicate determinations ± SD.

^e^ Light scattering assay. Data are the average of triplicate determinations ± SD.

### Cytotoxicity assays

The anti-migratory acridinones **6**, **7**, **9** and **10** were evaluated for their cytotoxic effects against MDA-MB-231 and DU-145 cancer cell lines (Table 1). The four compounds exhibited good potency with no significant differences between the two cell lines. As can be seen, compounds **7** and **10** had the highest potency (0.190 and 0.110 μM on MDA-MB-231, respectively), which was in agreement with the results of the migration assays. To assess the selectivity of these compounds, their cytotoxic effects were evaluated in normal fibroblasts (FGH) (Table 1). We were not able to determine their IC_50_ values because of solubility issues at concentrations above 200 μM.

**Fig 4:**
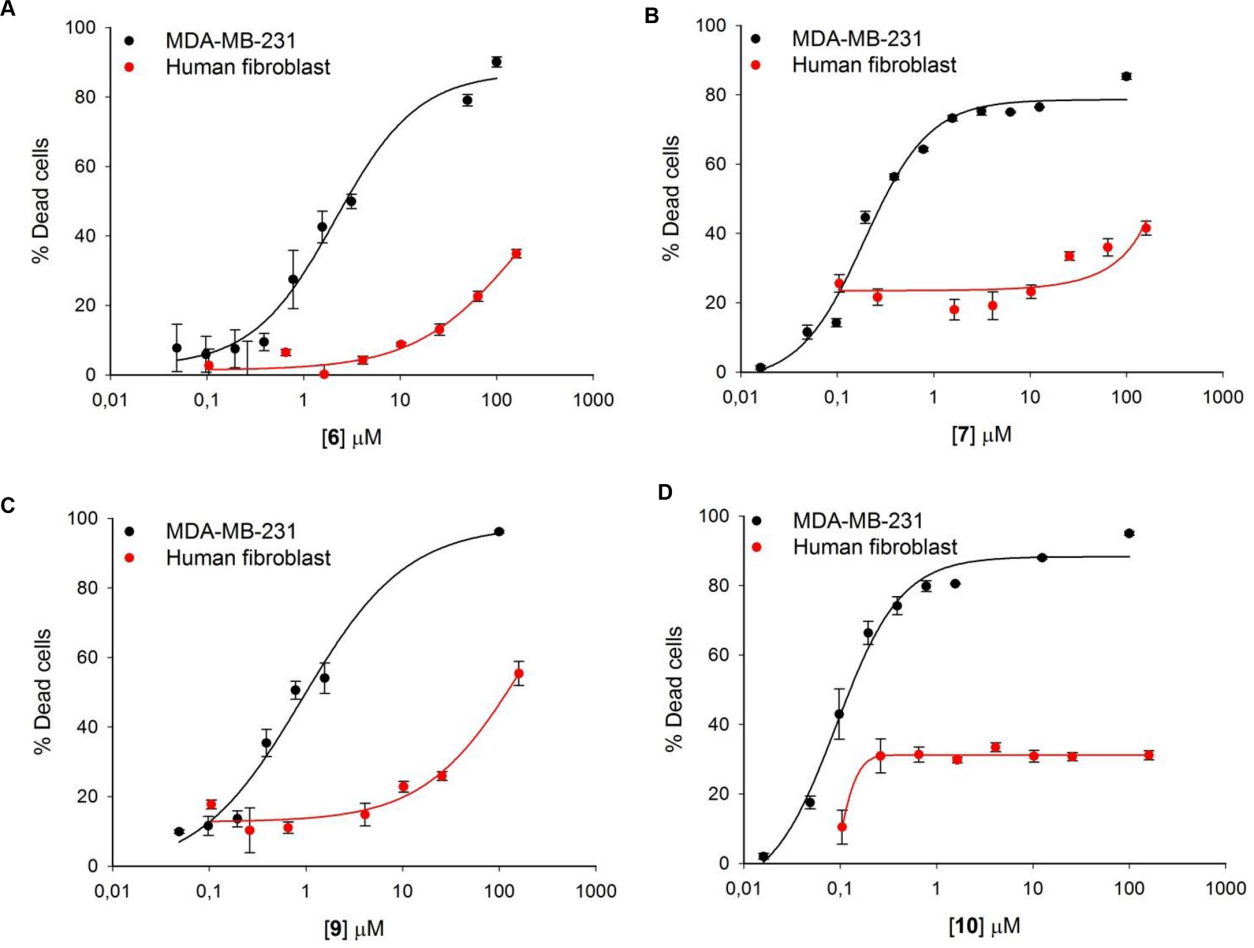
Comparison of cytotoxicity in MDA-MB-231 (black lines and circles) and FGH (red lines and circles) cells. The results for **6** (A), **7** (B), **9** (C), **10** (D). The mean ± SD of two independent experiments in triplicate is shown, and the percentage of dead cells is relative to negative control.

In a further attempt to understand the selectivity of the compounds, we evaluated them using two cell lines with similar concentrations (Fig 4). In all the tested concentrations, the compounds were more potent in the malignant MDA-MB-231 cell line by a factor > 30 compared to normal FGH cells (Table 1). In addition, we employed concentrations around the IC_50_ values in MDA-MB-231 cells (Table 1) to investigate the effects of the compounds in the FGH cells. Only 3, 20, 13 and 12% of cell death was obtained for compounds **6**, **7**, **9** and **10**, respectively. This revealed an interesting selectivity profile of these acridinones, considering that colchicine affects normal cells in a proportion comparable to that with cancer cells (Table 1). These results confirm the well-known high cytotoxicity of colchicine, which has restricted its therapeutic value for the treatment of cancer.

### Tubulin polymerization

**Fig 5:**
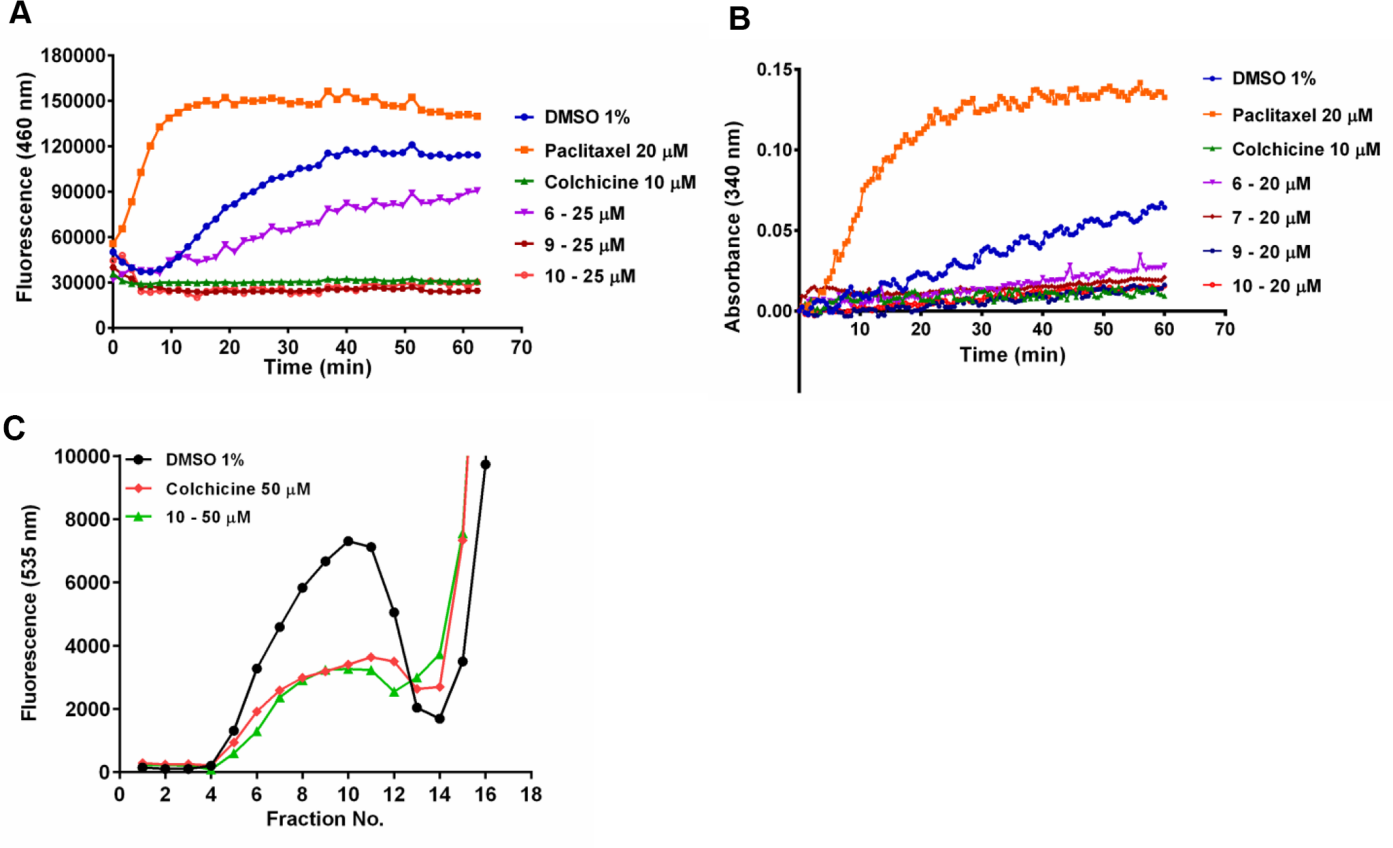
*In vitro* tubulin assembly inhibition. (A), results for the fluorescence-based assay; blue lines and circles represent the negative control (DMSO 1%), orange lines and squares represent the stabilizing control (paclitaxel) and green lines and triangles represent inhibition by the control (colchicine). **6** results are presented as purple lines and inverted triangles, **7** are dark red lines and hexagons and the results for **10** are presented as light red lines and circles (B), results for the light-scattering assay negative control, paclitaxel, colchicine, **6**, **7** and **10** are represented by the same colors and shapes as in (A), **9** is presented as dark blue lines and hexagons (C), competitive colchicine binding assay; black lines and circles represent the fluorescence of the negative control (DMSO 1%) as competitor, and the results for positive control colchicine as competitor are presented as red lines and diamonds; fluorescence in the presence of **10** as competitor is presented as green lines and triangles. Data are representative of two experiments. *To monitor a non-competitive colchicine-binding behavior, experiments using an inactive compound from our *in house* collection of compounds were conducted (data not shown).

The four acridinones inhibited tubulin assembly (Fig 5A and 5B), as predicted in our initial molecular modeling studies. The inhibitory properties of these compounds were assessed employing two distinct methodologies. First, we measured the increase in fluorescence as microtubules were polymerized *in vitro*, and then we confirmed the effects measuring the increases in light scattering. IC_50_ values were determined (Table 1), and again **7** and **10** were the most promising of the series (IC_50_ values of 2.4 and 0.9 μM, respectively), exhibiting potency comparable to that of colchicine. These findings strengthen the proposition that the cellular effects are due, at least in part, to tubulin modulation.

### Colchicine-site competitive assays

The four active acridinones have shown their ability to inhibit tubulin polymerization. Hence, it was essential to determine that the depolymerization properties of these compounds are associated with their binding to the colchicine-binding site of tubulin. For this purpose, we carried out a series of quantitative competitive assays using fluorescently labeled colchicine to characterize the binding of the acridinones to tubulin. As expected, excess of unlabeled colchicine (50 μM) inhibited 70% of fluorescein-labeled colchicine binding relative to the vehicle control. Compound **10**, at a concentration of 50 μM, behaved similarly (Fig 5C), inhibiting 72% of fluorescein-labeled colchicine binding. This shows that **10** binds to the colchicine-binding site of tubulin, confirming the results obtained in our molecular docking studies.

### Cell-cycle analysis

**Fig 6:**
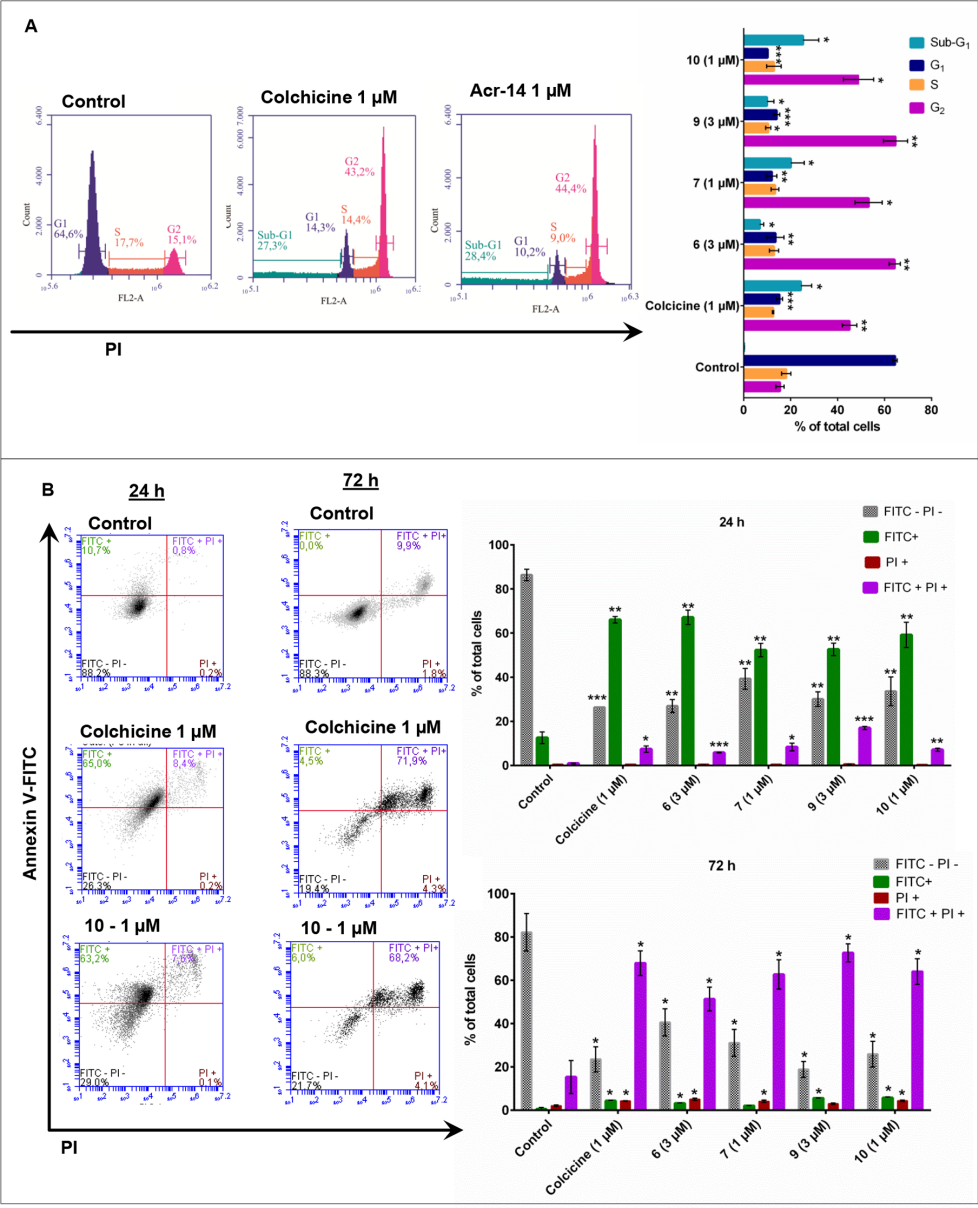
Flow cytometry analyses. (A), the left panel presents the fluorescence histogram with the DNA profile of the cell-cycle phases (Sub-G_1_, G_1_, S and G_2_) of MDA-MB-231 cells in the presence of the control (DMSO 0.1%), colchicine and **10**. The right panel shows the distribution of cells in different phases of the cell cycle for the control and after treatment with colchicine, **6**, **7**, **9** and **10** (B), the left panel displays the dot plot for the Annexin-V FITC/PI double stain of MDA-MB-231 cells for the control and after treatment with colchicine and **10**. In the FITC- PI- quadrant are the alive cells, in the FITC+ quadrant are the apoptotic cells, and in the FITC+ PI+ and PI+ quadrants are the dead cells. The right panel shows the percentage of alive, apoptotic and dead cells of the control and after treatment with colchicine, **6**, **7**, **9** and **10**. Statistical significance relative to control * *P* <0.05, ** *P* <0.01 and *** *P* <0.001.

Microtubule-interacting agents have been shown to arrest cells in G_2_/M as a result of mitotic-spindle disruption [39]. A synchronized culture of MDA-MB-231 was exposed to colchicine, **6**, **7**, **9** and **10** for 48 h (approximately 1.2 doubling time) and had their DNA content analyzed by flow cytometry. The results showed that all the compounds led to the accumulation of cells in the G_2_/M phase with a reduction in the number of cells in G_1_ relative to the vehicle control (Fig 6A). While colchicine arrested 42.9% of cells in G_2_, **6**, **7**, **9** and **10** arrested 53.6%, 49.1%, 53.6% and 44.4% of the cells, respectively. These results show that colchicine and the acridinones behave as mitotic arresters, which is consistent with the mechanism of action in microtubules, suggesting the *in situ* activity of tubulin.

### Apoptosis analysis

The effect of cell-cycle progression caused by microtubule inhibitors triggers signaling pathways such as mitotic spindle assembly checkpoint and Bcl-2 phosphorylation, leading cells to apoptosis [40, 41]. To investigate the ability of this series of acridinones to induce apoptosis in MDA-MB-231 cells, we evaluated the externalization of phosphatidylserine (PS), a hallmark of early apoptosis, after 24 and 72 h of treating the cells with the compounds. After 24 h, 10.7% of the vehicle control cells were apoptotic, whereas 69.4%, 54.2%, 54.6% and 63.2% of the cells treated with **6**, **7**, **9** and **10**, respectively, were apoptotic. Similarly, colchicine induced 65% of cells to apoptosis (Fig 6B). Thus, the acridinones induced cells to apoptosis or mitotic catastrophe [42], consistent with the proposed mechanism of action on microtubules.

As depicted in Fig 6B, most of cells were dead 72 h after the cultures were treated with the acridinones **6**, **7**, **9**, **10** and colchicine (55%, 67%, 75%, 68% and 72%, respectively), but only approximately 10% of the cells in the vehicle control were dead. These percentages of dead cells from apoptosis analyses were in the same order of magnitude as those determined at similar concentrations in MTS studies, in which 51%, 69%, 71% and 79% of cells were dead after treatment with compounds **6**, **7**, **9** and **10**, respectively. This independently confirmed the cytotoxicity results.

## Discussion

Tubulin is a valid drug target for cancer therapy, reflecting the success of the taxanes (e.g., paclitaxel, docetaxel) and vinca alkaloids (e.g., vinblastine, vincristine, and vinorelbine). However, development of new microtubule-interacting agents is needed because drugs that target microtubules are not efficient for treating all cancers and their efficacy has been limited by the development of drug resistance.

Although the natural alkaloid colchicine is a potent tubulin assembly inhibitor, it has limited therapeutic value because of its high toxicity. Other potent colchicine-binding site ligands, such as podophyllotoxin, have similar disadvantages. However, considerable research efforts have been made toward the discovery of new chemical entities (NCEs) to treat cancers that target the colchicine-binding site. One of the main advantages is the fact that its ligands are structurally smaller and simpler than those of the paclitaxel- and vinblastine-binding sites (e.g., taxanes and vinca alkaloids, respectively). These compounds interfere in cell migration and can help stop metastasis, which is another important benefit considering that metastasis is responsible for death in 90% of cancer cases [43–46]. They can also disrupt tumor vasculature, resulting in hypoxia-driven tumor necrosis [47]. Many known CBSI agents present antiangiogenic properties due to their interference in many functions of endothelial cells involved in angiogenesis, including cell motility [7, 48–50].

Our lead discovery strategy, inspired by the structure of podophyllotoxin, led to the identification of acridinones **6**, **7**, **9** and **10** as novel microtubule-assembly inhibitors possessing much simpler structural frameworks. This new series of compounds also inhibited cellular migration and exhibited potent cytotoxic activity against the human cancer cell lines MDA-MB-231 and DU-145. In addition, these compounds presented low toxicity against human normal FGH cells (highly selective), contrary to what was observed with colchicine, which kills normal and cancer cells in the same proportion (no selectivity). Consequently, the therapeutic indexes of these compounds are considerably higher than that of colchicine.

Our early molecular modeling investigations indicated that these molecules could interact with the colchicine-binding site, triggering the disruption of tubulin polymerization. These properties were experimentally confirmed through biochemical assays. Analyses of the DNA content by flow cytometry showed that cells were arrested in G_2_/M phase, affecting the mitosis process. This was accompanied by an increase in sub-G_1_ population, indicating induction of apoptosis, which was confirmed by phosphatidylserine externalization analysis.

In summary, we have developed a highly interesting series of acridinones that are capable of modulating two essential targets: a molecular target (the protein tubulin) and a phenotypic target (cell migration). Modulation of these targets can be related to promising cytotoxic effects in cancer cells with minor cytotoxicity for normal cells. These results show the significance of using natural products as starting points for the generation of simple lead compounds and their further development as novel anticancer agents.

## Supporting Information

S1 Appendix Synthetic procedures and compound characterization. (DOCX)

S1 Fig Structures of the tubulin target agents: colchicine, paclitaxel, vinblastine, vincristine, combretastatin A-4 and podophyllotoxin. (PDF)

S2 Fig Synthesis and drug design approach. (PDF)
