# Supplementary material for: Discovery of a Series of Acridinones as Mechanism-Based Tubulin Assembly Inhibitors with Anticancer Activity
Source: PLoS One. 2016 Aug 10;11(8):e0160842. doi: 10.1371/journal.pone.0160842 (PMC4980028; doi:10.1371/journal.pone.0160842)
Supplement: S1 Appendix — (DOCX) [file pone.0160842.s001.docx]

**S1 Appendix. Synthetic procedures and compound characterization.**

**General method for the multicomponent synthesis of acridinones 1 to 15.**

A mixture of *α*-naphthylamine or 3,4-(methylenedioxo)aniline (1 mmol), 1,3-cyclohexanedione (1 mmol) and a corresponding aldehyde (1 mmol) in ethanol (5 mL) was refluxed for 18-24 hours. The reaction was allowed to cool to room temperature, and the precipitated product was collected by vacuum filtration and washed with cold ethanol (10 mL). The impurities were removed by boiling the precipitate in ethanol (2-3 mL) for 5 minutes, followed by cooling and vacuum filtration with cold ethanol and ethyl ether (10 mL each).

7-phenyl-9,10,11,12-tetrahydrobenzo[*c*]-acridin-8(7*H*)-one **1**: Aldehyde: benzaldehyde. Yield: 45%. ^1^H-NMR (400 MHz, DMSO-*d*6): 1.90 (m, 2H), 2.0 (m, 2H), 2.26 (m 1H), 2.72 (m, 1H), 2.92 (m, 1H), 5.25 (s, 1H), 7.06-7.32 (m, 5H), 7.46-7.58 (m, 2H), 7.82 (m, 1H), 8.48 (m, 1H), 9.34 (broad s, 1H). ^13^C-NMR: 21.5, 27.44, 37.25, 108.88, 121.39, 121.74, 122.62, 123.16, 126.20, 127.58, 128.62, 131.11, 132.77, 148.88, 152.22, 194.42. ESI-MS (M+H^+^): 326.1547, calculated: 326.1545.

7-(3-methoxyphenyl)-9,10,11,12-tetrahydrobenzo[*c*]-acridin-8(7*H*)-one **2**: Aldehyde: 3-methoxybenzaldehyde. Yield: 30%. ^1^H-NMR (400 MHz, DMSO-*d*6): 2.02 (m, 2H), 2.28 (m, 2H), 2.72 (m, 1H), 2.92 (m, 1H), 3.67 (s, 3H), 5.23 (s, 1H), 6.64 (m, 1H), 6.80 (m, 2H) 7.10 (m, 1H), 7.36 (m, 1H) 7.47-7.59 (m, 3H), 7.83 (m, 1H), 8.50 (m, 1H), 9.35 (s, 1H). ^13^C-NMR: 21.55, 27.43, 37.26, 55.26 108.75, 110.91, 112.78, 113.85, 121.28, 122.60, 123.13, 126.20, 128.33, 129.65, 131.07, 132.77, 150.36, 159.55, 194.45. ESI-MS (M+H^+^): 356.1644, calculated: 356.1651.

7-(4-methoxyphenyl)-9,10,11,12-tetrahydrobenzo[*c*]-acridin-8(7*H*)-one **3** Aldehyde:. Yield: 25% Melting point: 209 ^o^C. ^1^H-NMR (400 MHz, DMSO-*d*6): 1.91 (m, 2H), 1.98 (m, 2H), 2.25 (m, 2H), 2.68 (m, 1H), 2.90 (m, 1H), 3.64 (s, 3H), 5.19 (s, 1H), 6.73 (m, 3H), 6.96 (m, 1H), 7.12 (m, 2H), 7.29 (m, 1H), 7.45-7.58 (m, 3H), 7.82 (m, 1H), 8.48 (m, 1H), 9.31 (broad s, 1H). ^13^C-NMR: 21.54, 27.43, 37.27, 55.37, 109.19, 113.95, 121.70, 122.62, 123.10, 126.10, 128.53, 131.02, 132.72, 141.31, 153.93, 157.81, 192.42. ESI-MS (M+H^+^): 356.1645, calculated: 356.1651.

7-(3-hydroxyphenyl)-9,10,11,12-tetrahydrobenzo[*c*]-acridin-8(7*H*)-one **4**: Aldehyde: 3-hydroxybenzaldehyde. Yield: 8% Melting Point: 300 ^o^C (decomp.) ^1^H-NMR (400 MHz, DMSO-*d*6): 1.82-1.93 (m, 2H), 2.18 (m, 2H), 2.59 (m, 2H). 5.23 (s, 1H), 5.88 (m, 1H), 5.95 (m, 1H), 6.57 (m, 1H), 6.81 (m, 1H), 7.50 (m, 1H), 7.64 (m, 1H), 7.95 (m, 1H), 8.01 (m, 1H), 9.51 (broad s, 1H). ^13^C-NMR: 21.49, 27.41, 37.01, 97.28, 101.46, 106.22, 109.37, 117.30, 121.27, 121.83, 130.15, 130.85, 134.37, 143.74, 146.83, 148.15, 151.21, 154.28, 193.75. ESI-MS (M+H^+^): 342.1486, calculated: 342.1494.

7-(4-hydroxyphenyl)-9,10,11,12-tetrahydrobenzo[*c*]-acridin-8(7*H*)-one **5**: Aldehyde: 4-hydroxybenzaldehyde. Yield: 11%. Melting point: 249-253 ^o^C. ^1^H-NMR (400 MHz, DMSO-*d*6): 1.92 (m, 2H), 2.27 (m, 2H), 2.71 (m, 1H), 2.91 (m, 1H), 5.15 (s, 1H), 6.59 (m, 2H), 7.01 (m, 2H), 7.31 (m, 1H), 7.49 (m, 3H), 7.83 (m, 1H), 8.48 (m, 2H), 9.11 (s, 1H), 9.29 (s, 1H). ^13^C-NMR: 21.59, 27.51, 37.37, 109.38, 115.26, 115.29, 115.31, 121.74, 122.03, 122.67, 123.08, 126.09, 126.16, 128.52, 128.69, 131.73, 132.03, 139.72, 153.88, 155.89, 194.47. ESI-MS (M+H^+^): 342.1481, calculated: 342.1484.

7-(benzo[*d*][1,3]dioxol-5-yl)-9,10,11,12-tetrahydrobenzo[*c*]-acridin-8(7*H*)-one **6**: Yield. 51%. Melting point: 154 ^o^C (decomp.). ^1^H-NMR (400 MHz, DMSO-*d*6): 1.93 (m, 2H), 2.28 (m, 2H), 2.73 (m, 1H), 2.92 (m, 1H), 5.19 (s, 1H), 5.89 (m, 2H), 6.71 (m, 4H), 7.34 (m, 1H), 7.50 (m, 3H), 7.84 (m, 1H), 8.48 (m, 1H), 9.33 (s, 1H). ^13^C-NMR: 21.56, 27.46, 37.29, 101.07, 108.18, 108.32, 109.07, 119.89, 120.41, 121.50, 121.76, 122.65, 123.17, 126.18, 128.36, 128.68, 131.02, 132.79, 143.27, 145.70, 147.15, 147.52, 154.09, 194.47. ESI-MS (M+H^+^): 370.1439, calculated: 370.1443.

7-(3-nitrophenyl)-9,10,11,12-tetrahydrobenzo[*c*]-acridin-8(7*H*)-one **7** : Yield. 32%. Melting point: 153 ^o^C (decomp.). ^1^H-NMR (400 MHz, DMSO-*d*6): 1.92 (m, 2H), 2.28 (m, 2H), 2.76 (m, 1H), 2.95 (m, 1H), 5.49 (s, 1H), 7.52 (m, 5H), 7.85 (m, 1H), 7.96 (m, 1H), 8.09 (m, 1H), 8.52 (m, 1H), 9.49 (s, 1H). ^13^C-NMR: 21.54, 27.46, 37.16, 107.60, 108.23, 120.12, 121.47, 121.87, 122.06, 122.67, 123.63, 126.44, 126.52, 128.21, 128.76, 131.32, 133.01, 134.63, 148.22, 150.87, 154.81, 194.51. ESI-MS (M+H^+^): 371.1383, calculated: 371.1396.

4-(8-oxo-7,8,9,10,11,12-hexahydrobenzo[c]acridin-7-yl)benzoic acid **8**: Yield. 13%. Melting point: 280 ^o^C (decomp.). ^1^H-NMR (400 MHz, DMSO-d6): 1.89-2.00 (m, 2H), 2.27 (m, 2H), 2.75 (m, 1H), 2.92 (m, 1H), 5.35 (s, 1H), 7.37 (m, 3H), 7.50-7.60 (m, 3H), 7.78-7.75 (m, 3H), 8.50 (m, 1H), 9.40 (s, 1H), 12.74 (broad s, 1H). ^13^C-NMR: 21.49, 27.44, 37.20, 108.39, 120.58, 121.80, 122.65, 123.34, 126.30, 126.34, 127.84, 128.25, 128.70, 128.81, 129.87, 131.19, 132.89, 153.62, 154.50, 167.66, 194,41. ESI-MS (M+H^+^): 370.1446, calculated: 370.1443.

7-(2-thiophenyl)-9,10,11,12-tetrahydrobenzo[c]-acridin-8(7H)-one **9**: Yield. 32%. Melting point: 255 ^o^C (decomp.). ^1^H-NMR (400 MHz, DMSO-d6): 2.02 (m, 2H), 2.23 (m, 2H), 2.72 (m, 1H), 2.90 (m, 1H), 5.55 (s, 1H), 6.73 (m, 2H), 6.81, (m, 2H), 7.17 (m, 1H), 7.41-7.55 (m, 5H), 7.87 (m, 1H), 8.50 (m, 1H), 9.50 (s, 1H). ^13^C-NMR: 21.51, 27.40, 35.43, 37.22, 108.63, 120.62, 121.77, 122.60, 123.09, 123.34, 124.19, 126.33, 127.02, 128.25, 128.73, 131.20, 132.96, 153.09, 154.24, 194.34. ESI-MS (M+H^+^): 332.1098, calculated: 332.1108.

7-(3,4,5-trimethoxyphenyl)-9,10,11,12-tetrahydrobenzo[*c*]-acridin-8(7*H*)-one **10**: Yield. 28%. Melting point: 280 ^o^C. ^1^H-NMR (400 MHz, DMSO-*d*6): 1.96 (m, 2H), 2.31 (m, 2H), 2.75 (m, 1H), 2.97 (m, 1H), 3.57 (s, 3H), 3.70 (s, 6H), 5.22 (s, 1H), 6.57 (m, 2H), 7.50 (m, 5H), 7.83 (m, 1H), 8.47 (m, 1H), 9.35 (s, 1H). ^13^C-NMR: 21.74, 27.54, 37.41, 56.24, 60.32, 104.32, 108.55, 121.49, 121.78, 122.67, 123.11, 126.18, 128.33, 128.67, 130.91, 132.79, 136.29, 144.58, 153.10, 154.43, 194.55. ESI-MS (M+H^+^): 416.1870, calculated: 416.1862.

10-(3-methoxyphenyl)-6,7,8,10-tetrahydro-[1,3]dioxolo[4,5-*b*]acridin-9(5*H*)-one **11**: Yield. 52%. Melting point: 242 ^o^C. ^1^H-NMR (400 MHz, DMSO-*d*6): 1.85 (m, 2H), 2.20 (m, 2H), 2.59 (m, 2H), 3.69 (s, 3H), 5.00 (s, 1H), 5.88 (s, 1H), 5.94 (m, 1H), 6.54 (m, 1H), 6.65 (m, 1H) 6.67 (m, 3H), 7.10 (m, 1H), 9.33 (s, 1H). ^13^C-NMR: 21.58, 27.48, 37.22, 39.94, 53.30, 97.70, 101.29, 106.87, 109.31, 110.82, 113.65, 118.64, 119.72, 129.56, 130.85, 143.44, 146.45, 150.76, 153.78, 159.57, 193.65. ESI-MS (M+H^+^): 350.1390, calculated: 350.1392.

10-(4-methoxyphenyl)-6,7,8,10-tetrahydro-[1,3]dioxolo[4,5-*b*]acridin-9(5*H*)-one **12**: Yield. 45%. Melting point: 242 ^o^C (decomp.). ^1^H-NMR (400 MHz, DMSO-*d*6): 1.83 (m, 2H), 2.18 (m, 2H), 2.57 (m, 2H), 3.67 (s, 3H), 4.97 (s, 1H), 5.87 (m, 2H), 5.94 (m, 2H), 6.53 (m, 1H), 6.76 (m, 4H), 7.06 (m, 2H), 9.32 (s, 1H). ^13^C-NMR: 21.55, 27.46, 37.22, 39.05, 55.41, 97.02, 101.26, 107.27, 109.30, 119.12, 128.29, 130.80, 141.64, 143.40, 146.30, 153.52, 193.66. ESI-MS (M+H^+^): 350.1396, calculated: 350.1392.

10-(3-methoxy-4-hydroxyphenyl)-6,7,8,10-tetrahydro-[1,3]dioxolo[4,5-*b*]acridin-9(5*H*)-one **13**: Yield. 30%. Melting point: 293 ^o^C (decomp.). ^1^H-NMR (400 MHz, DMSO-*d*6): 1.85 (m, 2H), 2.20 (m, 2H), 2.57 (m, 2H), 3.71 (s, 3H), 4.91 (s, 1H), 5.87 (m, 1H), 5.93 (m, 1H), 6.51 (m, 2H), 6.56 (m, 1H), 6.79 (m, 2H), 8.62 (s, 1H), 9.28 (s, 1H). ^13^C-NMR: 21.62, 27.49, 37.28, 39.29, 56.04, 96.96, 101.22, 107.20, 109.30, 111.86, 115.61, 119.36, 130.70, 140.59, 143.34, 144.93, 146.22, 147.49, 153.56, 193.75. ESI-MS (M+H^+^): 366.1340, calculated: 350.1341.

10-phenyl-6,7,8,10-tetrahydro-[1,3]dioxolo[4,5-*b*]acridin-9(5*H*)-one **14**: Yield. 84%. Melting point: 280 ^o^C (decomp.). ^1^H-NMR (400 MHz, DMSO-*d*6): 1.85 (m, 2H), 2.19 (m, 2H), 2.59 (m, 2H), 5.04 (s, 1H), 5.87 (m, 1H), 5.94 (m, 1H), 6.55 (m, 1H), 6.75 (m, 1H), 7.07 (m, 1H), 7.18 (m, 4H), 9.36 (s, 1H). ^13^C-NMR: 21.54, 27.47, 37.19, 97.08, 101.30, 106.96, 109.32, 118.76, 126.06, 127.38, 128.56, 130.85, 143.45, 146.42, 149.25, 153.81, 193.67. ESI-MS (M+H^+^): 320.1280, calculated: 320.1287.

10-(4-nitrophenyl)-6,7,8,10-tetrahydro-[1,3]dioxolo[4,5-*b*]acridin-9(5*H*)-one **15**: Yield: 15% Melting Point: 250 ^o^C (decomp.). ^1^H-NMR (400 MHz, DMSO-*d*6): 1.83 (m, 2H), 1.93 (m, 2H), 2.18 (m, 4H), 2.58 (m, 2H), 5.20 (s, 1H), 5.88 (s, 1H), 5.95 (s, 1H), 6.56 (m, 1H), 6.75 (m, 1H), 7.45 (m, 2H), 8.09 (m, 2H), 9.50 (s, 1H). ^13^C-NMR: 21.44, 27.43, 37.01, 97.32, 101.47, 106.02, 109.33, 117.04, 123.97, 128.62, 130.84, 143.69, 146.02, 146.86, 152.25, 156.49, 193.64. ESI-MS (M+H^+^): 365.1148, calculated: 365.1187.
