# Supplementary material for: Discovery of a Series of Acridinones as Mechanism-Based Tubulin Assembly Inhibitors with Anticancer Activity
Source: PLoS One. 2016 Aug 10;11(8):e0160842. doi: 10.1371/journal.pone.0160842 (PMC4980028; doi:10.1371/journal.pone.0160842)
Supplement: S1 Fig — (PDF) [file pone.0160842.s002.pdf]

S1 Fig. Structures of the tubulin target agents: colchicine, paclitaxel, vinblastine, vincristine, combretastatin A-4 and podophyllotoxin.

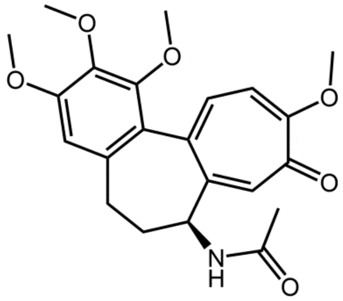

Colchicine

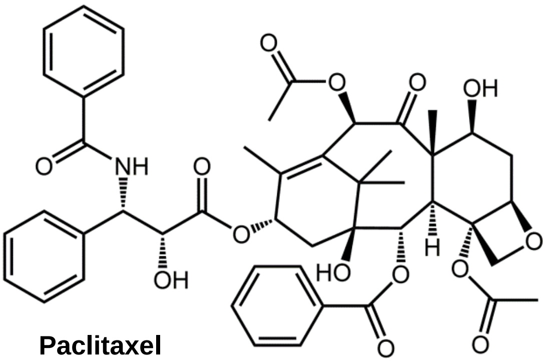

Paclitaxel

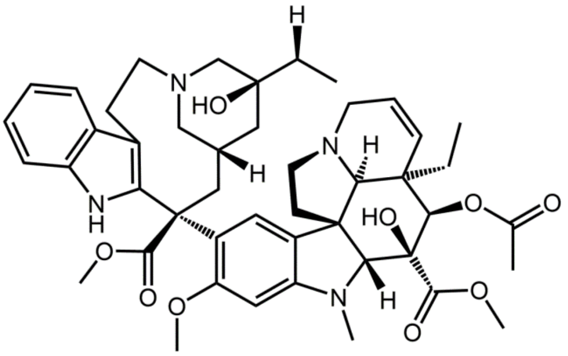

Vinblastine

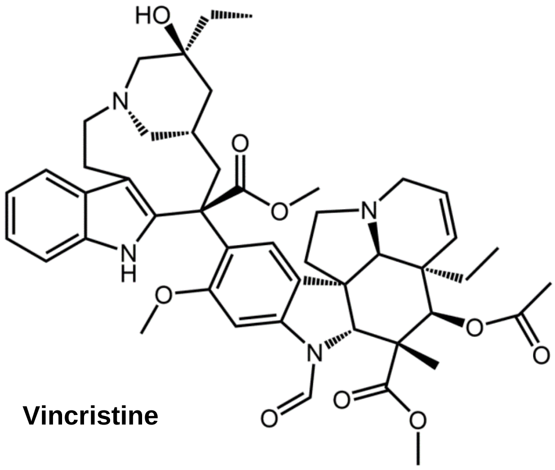

Vincristine

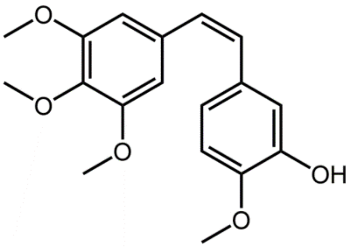

Combretastatin A-4

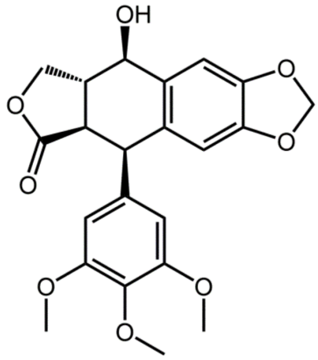

Podophyllotoxin
