# Supplementary figures and images for: Discovery of a Series of Acridinones as Mechanism-Based Tubulin Assembly Inhibitors with Anticancer Activity
Source: PLoS One. 2016 Aug 10;11(8):e0160842. doi: 10.1371/journal.pone.0160842 (PMC4980028; doi:10.1371/journal.pone.0160842)

**S2 Fig. Synthesis and drug design approach.**

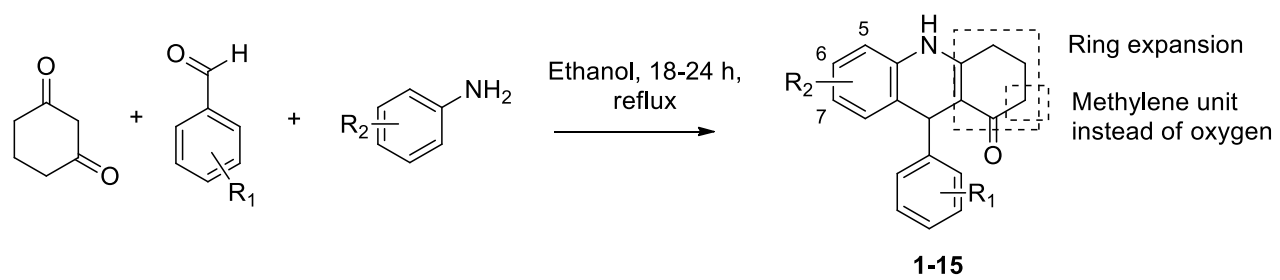

Supplement: S2 Fig — (PDF) [file pone.0160842.s003.pdf]
